# Effectiveness of integrative medicine group visits in chronic pain and depressive symptoms: A randomized controlled trial

**DOI:** 10.1371/journal.pone.0225540

**Published:** 2019-12-18

**Authors:** Paula Gardiner, Man Luo, Salvatore D’Amico, Katherine Gergen-Barnett, Laura F. White, Robert Saper, Suzanne Mitchell, Jane M. Liebschutz

**Affiliations:** 1 Department of Family Medicine, University of Massachusetts Medical School, Worcester, Massachusetts, United States of America; 2 Department of Family Medicine, Boston University School of Medicine and Boston Medical Center, Boston, Massachusetts, United States of America; 3 Department of Biostatistics, Boston University School of Public Health, Boston, Massachusetts, United States of America; 4 Division of General Internal Medicine, University of Pittsburgh School of Medicine, Pittsburgh, Pennsylvania, United States of America; Brown University, UNITED STATES

## Abstract

**Background:**

Current treatment options for chronic pain and depression are largely medication-based, which may cause adverse side effects. Integrative Medical Group Visits (IMGV) combines mindfulness techniques, evidence based integrative medicine, and medical group visits, and is a promising adjunct to medications, especially for diverse underserved patients who have limited access to non-pharmacological therapies.

**Objective:**

Determine the effectiveness of IMGV compared to a Primary Care Provider (PCP) visit in patients with chronic pain and depression.

**Design:**

9-week single-blind randomized control trial with a 12-week maintenance phase (intervention—medical groups; control—primary care provider visit)

**Setting:**

Academic tertiary safety-net hospital and 2 affiliated federally-qualified community health centers.

**Participants:**

159 predominantly low income racially diverse adults with nonspecific chronic pain and depressive symptoms.

**Interventions:**

IMGV intervention– 9 weekly 2.5 hour in person IMGV sessions, 12 weeks on-line platform access followed by a final IMGV at 21 weeks.

**Measurements:**

Data collected at baseline, 9, and 21 weeks included primary outcomes depressive symptoms (Patient Health Questionnaire 9), pain (Brief Pain Inventory). Secondary outcomes included pain medication use and utilization.

**Results:**

There were no differences in pain or depression at any time point. At 9 weeks, the IMGV group had fewer emergency department visits (RR 0.32, 95% CI: 0.12, 0.83) compared to controls. At 21 weeks, the IMGV group reported reduction in pain medication use (Odds Ratio: 0.42, CI: 0.18–0.98) compared to controls.

**Limitations:**

Absence of treatment assignment concealment for patients and disproportionate group attendance in IMGV.

**Conclusion:**

Results demonstrate that low-income racially diverse patients will attend medical group visits that focus on non-pharmacological techniques, however, in the attention to treat analysis there was no difference in average pain levels between the intervention and the control group.

**Trial registration:**

clinicaltrials.gov NCT02262377.

## Introduction

Chronic pain annually affects 25 million adults in the United States and is linked to significant disability and high medical utilization [1–3]. Treatment of chronic pain is complex due to safety concerns of prescription pain medications (e.g. opioids) and comorbid conditions such as depression and substance use [4,5]. Depression often complicates the treatment of chronic pain [6,7]. Even when pain medications are effective in reducing pain; they may not improve mental and functional status and may actually increase depression [8,9]. Furthermore, patients with chronic pain and depressive symptoms have increased use of medical care and higher risk of medical utilization [10–12].

Due to socioeconomic factors, treating chronic pain and depressive symptoms may be challenging in a low income, racially and ethnically diverse patients [13,14]. Disparities in access to prescription and non-prescription treatment for chronic pain and associated conditions may contribute to negative impact on economic (ability to work), emotional (social isolation), and daily functioning [15–17]. For example, minority patients with chronic pain receive less patient education, medications, surgery, and specialty referrals [14]. Access to non-pharmacological therapies is challenging as these therapies are often located far from low income neighborhoods, require an out of pocket payment, may not be covered by health insurance, and are less likely to be offered as a treatment to low-income or under-represented minority patients [18–23].

One such non-pharmacological treatment is Evidence Based Integrative Medicine (EBIM) which combines “mainstream medical therapies and complementary therapies for which there is high-quality scientific evidence of safety and effectiveness” [24,25]. EBIM addresses factors such as activity, social connection, nutrition, lifestyle modification, and stress, all of which play significant roles in chronic conditions [26]. Clinical studies on EBIM demonstrate health improvements in patients’ chronic pain and/or depressive symptoms [27]. For example, mind body techniques, such as Mindfulness Based Stress Reduction (MBSR), demonstrate benefits for individuals with chronic pain [8,9,28]. Several systematic reviews on mindfulness clinical trials for patients with chronic pain show improvement in pain scores and mental health status [5, 29–32].

In 2012, at Boston Medical Center, an urban safety-net hospital, the Integrative Medical Group Visit (IMGV) was developed to increase access to EBIM for patients in the outpatient setting. IMGV combines a medical group visit (MGV), principles of mindfulness, and EBIM [33,34]. We chose to use the medical group visit as the means to deliver EBIM for the following reasons: clinicians can bill insurance for the medical group visit, it increases access to EBIM, patients were introduced to EBIM in a trusted environment, and the medical group visits could be conducted in local neighborhood community health centers affiliated with BMC [35–37]. Medical group visits (shared medical appointments) are comprised of two clinicians who treat a group of eight to twelve patients at one time and include: individual medical attention, patient education, self-management, self-monitoring, and social support. MGVs are used to treat an increasing number of chronic illnesses and improve symptom management [38]. Current literature suggests that MGVs improve health-related quality of life, patient satisfaction, and reduce health care utilization [39,40].

In an uncontrolled study, the IMGV model demonstrated the potential for reducing pain and depressive symptoms [40]. Additionally, it was found to increase quality of life and reduce Emergency Department (ED) use [33]. However, it is unknown how the IMGV compares to a Primary Care Provider (PCP) visit in socioeconomically diverse patients with chronic pain and depressive symptoms [33,41–43]. This paper reports the main outcome findings of a single blind randomized controlled trial comparing the effectiveness of an IMGV in reducing pain, depressive symptoms, and pain medication use to a primary care visit. Our primary hypothesis was a greater reduction in pain, depressive symptoms, and medication use for participants randomized to IMGV compared with participants randomized to the control group. Additionally, this analysis examines who attended the IMGV and correlates of high versus low IMGV attendance.

## Materials and methods

The study was approved by the Boston University Medical Campus Institutional Review Board (IRB) and the community health center’s (CHC) research committees (IRB Approval Number: H33096). We registered this randomized controlled trial (RCT) in the international trial register [ClinicalTrials.gov: Identifier NCT02262377]. For further detail please refer to our methods paper [34].

This RCT was conducted at an ambulatory primary care clinic at the Boston Medical Center (BMC) and two affiliated federally qualified Community Health Centers (CHC): Codman Square Health Center (CSHC) and DotHouse Health (DH). These practices serve low-income, racially and ethnically diverse populations living in the Boston area. Our inclusion criteria included: age 18 years or older, able to communicate in English language, score of ≥ 5 on the Patient Health Questionnaire-9 (PHQ-9), score of ≥ 4 on a 0–10 scale measuring daily chronic pain intensity for at least 12 weeks, and having a PCP located at the site where the IMGV was being held [43–47]. The exclusion criteria included: self-reported symptoms of psychosis or mania, active substance abuse (alcohol, cocaine or heroin use in the last 3 months), previous participation in an IMGV, a new pain treatment in the past month or plans to begin any new pain treatments in the next three months, active suicidality, any other severe disabling chronic medical or psychiatric co-morbidities preventing attendance to the IMGV, or no access to the internet during the study period [34].

Participants were recruited through their clinicians’ outpatient referral, clinicians’ letter to patients about the study, or self-referral. After being contacted by the research assistant (RA), patients then consented to be screened. If the eligibility was verified and there was patient written consent, the patient was next enrolled in the study.

This study is a single-blinded, two-arm randomized controlled trial. All participants (N = 155) who were consented and completed baseline assessments were randomized (1:1) to either intervention (IMGV) or control group. A randomization list was created using computer-generated permuted blocks with a block size of 6. We used the Studytrax database system to designate the treatment assignments. These were placed in opaque, sequentially numbered, sealed envelopes, which were only opened by a research assistant when a new enrolled participant received their treatment allocation. The investigators and biostatisticians were blinded to the treatment assignment. Patients were not blinded to allocation due to the group nature of the intervention. All patients in the control group were offered to access to the IMGV groups after study completion.

### IMGV Intervention

The IMGV intervention includes three concurrent deliveries of the same self- management curriculum delivered with different formats–an in-person MGV, and two adjunct companion technologies available on a computer tablet provided to the intervention participant. The first technology was the Our Whole Lives (OWL), an e-Health toolkit platform, and the second technology was an Embodied Conversational Agent (ECA).

A detailed description of the IMGV self management intervention has previously been described [34]. The IMGV consists of a total of ten in-person medical group visits each lasting 2.5-hours conducted weekly from week 1 to week 9 (9 in-person sessions plus OWL/ECA). This is followed by a 12-week maintance phase where there is access to the technology only (OWL/ECA). A tenth and final in-person session is conducted at week 21.

At the start of the IMGV, participants measured their vital signs, moodstate, and pain levels. They then met individually with a trained physician (a co-facilatator) for a medical assessment. Two trained non-physician facilitators (see below) then led mindfulness practices. Patients were instructed in the principles of mindfulness and EBIM self-management techniques (such as acupressure and massage). Each week, the physician facilitated a discussion on health topics such as stress, insomnia, depression, chronic pain cycle, activity, and healthy food choices. Finally, the IMGV ended with a healthy meal, which mirrored the healthy nutrition topic in each session.

In addition to a physician, an experienced co-facilitator with training in mindfuness (certified MBSR instructor, yoga and meditation teacher) attended all groups. Facilitators were mentored via direct observation of two pilot group visits, one-on-one meetings, and phone calls by an experienced MBSR trained faculty.

To reinforce all content delivered in the in-person group, an internet-based platform called Our Whole Lives (OWL) delivered the same in-person curriculum. OWL could be accessed with a computer, smart phone, or tablet. The ECA, a female automated character, emulated the conversational behavior of an empathic coach [48]. The ECA (Gabby) reviewed all the content discussed in the IMGV with the participants outside of the in-person group. A Dell Venue 8 Pro tablet was distributed to all intervention participants in the first session of the group. Results of the use of technology will be published in an additional manuscript.

After the nine-week in-person group visit phase concluded, the intervention participants entered a 12-week maintenance phase. The intervention participants retained the study tablet and continued to have access to the ECA and the OWL website. At the end of the 21 weeks, there was one final in-person group visit.

A trained study RA directly observed all groups and assessed the facilitator’s adherence to the intervention components through a monitoring and evaluation checklist. These checklists were used to assess each MGV session at all sites during the study.

Prior to the start of the study, we provided continuing medical education training in evidence based chronic pain management at the study sites. We also provided access to the educational content on safe prescribing practices for chronic pain patients available on a website (http://mytopcare.org/) through small group presentations and/or Grand Round presentations at each study site for staff and clinical providers [49]. We did not collect data on who attended the training.

All participants randomized to the control group were asked to visit their PCP during the study period (baseline to 21 weeks). We verified a PCP clinical visit, via electronic medical record (EMR) documentation. We did not collect data on the duration or content of the visit.

### Outcomes

Research assistants collected outcome data at baseline, 9 weeks, and 21 weeks. Self-reported data included: baseline demographics (age, gender, race, ethnicity, income, work status, education) and types of pain medications used in the last seven days.

Our primary outcomes included: 1) self-reported pain measured by the Brief Pain Inventory [(BPI) pain interference, pain severity, and average pain score in the last seven days] [45,50] and 2) depression level measured by the PHQ-9, a self-reported depression scale [49,50]. BPI pain interference, pain severity, and average pain are on a 0-10-point scale. The higher the score, the more severe the pain. PHQ-9 is on a 0-27-point scale. The higher the score, the more severe the depression.

Secondary outcomes included pain self-efficacy, self-reported pain medication use, health-related quality of life, patient activation, risk of opioid misuse, and ED use [51–60]. Pain self-efficacy was measured with the Pain Self-Efficacy Scale (PSEQ) and ranged from 0–60 points. High PSEQ scores are associated with higher confidence to function with pain [61]. For self-reported pain medication use, we used the Timeline Follow Back method to determine patient reported use of pain medications in the prior seven days [51, 62]. We categorized pain medications by opioids (MS Contin, Vicodin, Oxycodone, OxyContin, Tramadol, Tylenol with Codeine #3), Nonsteroidal Anti-inflammatory Drugs (NSAIDS: Ibuprofen, Naproxen, Aspirin), and other pain medication (Acetaminophen, Cyclobenzaprine, Gabapentin).

Health-related quality of life was measured using the Short form 12 Health Survey version 2 (SF-12). The SF-12 is composed of two component scores: Mental Component Summary (MCS) and Physical Component Summary (PCS) [63]. SF-12 scores ranged from 0–100 points, where a zero score indicates the lowest level of health and 100 indicates the highest level of health. Activation in patients was measured using Patient Activation Measure (PAM) [52,55]. PAM scores are transformed to a scale of 0–100 points. The risk of opioid misuse was measured using the Common Opioid Misuse Measure (COMM) [58, 64]. COMM is a 17-item assessment measure and scored based on a Likert 5-point scale from 0–4. The COMM cut-off score of 9 or above is a positive indicator for misusing medication.

We measured ED utilization for the 12 weeks prior to the study and throughout the study period through self-reported ED use at baseline, 9 weeks, and 21 weeks and through the EMR. After completing the 9 and 21-week data collection, patients received a $25 gift card incentive.

We assumed a two-sided alpha error = 0.05 and estimated a 20% dropout rate from baseline to 21-weeks. Based on previous literature, we defined a statistically significant change in effect size to include reductions in average pain from the BPI (1.5 points) and PHQ-9 (4 points) [65,66]. Although some debate exists on how to define a minimal clinically important change, many pain researchers consider changes in pain of more than 1–1.5 points to be clinically important [67]. A sample size of 62 participants per treatment group across all sites had an 80% power to detect a 1.5 difference in average pain and a sample size of 31 per treatment group had 80% power to detect a difference of 4 points in PHQ-9 score. Additionally, we defined a clinical meaningful result as a 1.5 reduction in average pain or 4-point reduction in PHQ-9 score [65,66].

### Data analysis

We performed descriptive data analysis for baseline demographics. Means and standard deviations were calculated for continuous variables, and frequencies and percentages were calculated for categorical variables. To examine the success of randomization, we applied Pearson’s Chi-Square Test and Fisher’s Exact Test (categorical variable), and two-sample T-Test and Wilcoxon Rank-Sum Test (continuous variable) to compare the results between intervention and control at baseline, with a significance level of 0.1. Variables that were significantly different across study groups at baseline were considered confounders and were adjusted for in the following analysis. All data were analyzed using SAS 9.3. [68].

The primary analysis was intention-to-treat analysis. The ITT analysis included all participants who were randomized in the study, regardless of adherence to attending the IMGV or attending a PCP visit (control). To address missing data in all analyses, we used multiple imputation approach with 20 imputed data sets.

We performed the ITT analyses for the primary (pain and depressive symptoms) and secondary outcome variables. Descriptive statistics were calculated for all the outcome variables (Mean, SD, or N, %). Histograms were created to assess if the variables were normally distributed. Sensitivity analysis was performed on all multivariable regressions and logistic regressions.

For primary and secondary outcomes, we used multivariable regression models fit with generalized estimating equations (GEEs) to account for serially collected data, with an indicator for treatment assignment as the predictor of interest. We adjusted our models for potential confounders and assessed effect modification. For continuous and count variables we considered different regression models (Poisson, Negative Binomial, and Log Normal Model, as appropriate) to obtain the best fit for our outcomes. The models with lowest Akaike Information Criteria (AIC) were selected. Dichotomous outcomes (any pain medication use, opioid use, NSAIDS use, and other medication use) were fit with logistic regression. An interaction term of time and treatment was included in our models to assess for changes in the treatment over time.

On the advice of our patient advisory group and scientific advisory group, we conducted an exploratory per-protocol attendance analysis to understand how the “exposure to amount of health care”affected outcomes in those participants with no PCP visits during the study, low attendance to IMGV (1–4 sessions), medium attendance (5–6 sessions), and high attendance (7–10 sessions). We examined the baseline characteristics as well as longitudinal multivariable regressions, comparing intervention participants who attended different numbers of sessions to the control participants who did and did not attend PCP visits. The predictor of interest was treatment assignment, which indicated either the number of IMGV sessions attended (1–4 sessions, 5–6 sessions, and 7–10 sessions), or the control PCP visits (≥ 1 PCP visit, and no PCP visit). Poisson and negative binomial models were selected where AIC was minimized. Potential confounders were adjusted for in all models. A significance level of 0.05 was used, except where otherwise noted [68]. As we are performing multiple analyses, it is possible that we will see p-values that are below 0.05 by chance alone. Therefore, we do not strictly interpret statistical significance at the 0.05 level for analyses beyond the primary aims of the study and view these results as suggestive of areas that might merit further study.

Data on adverse events were reviewed and monitored on a quarterly basis by the PI. A Data Safety Monitoring Board (DSMB) reviewed all adverse events, data collection, and adherence to research protocol independently of study staff. All participants were included in the safety analyses using descriptive statistics.

## Results

**Fig 1:**
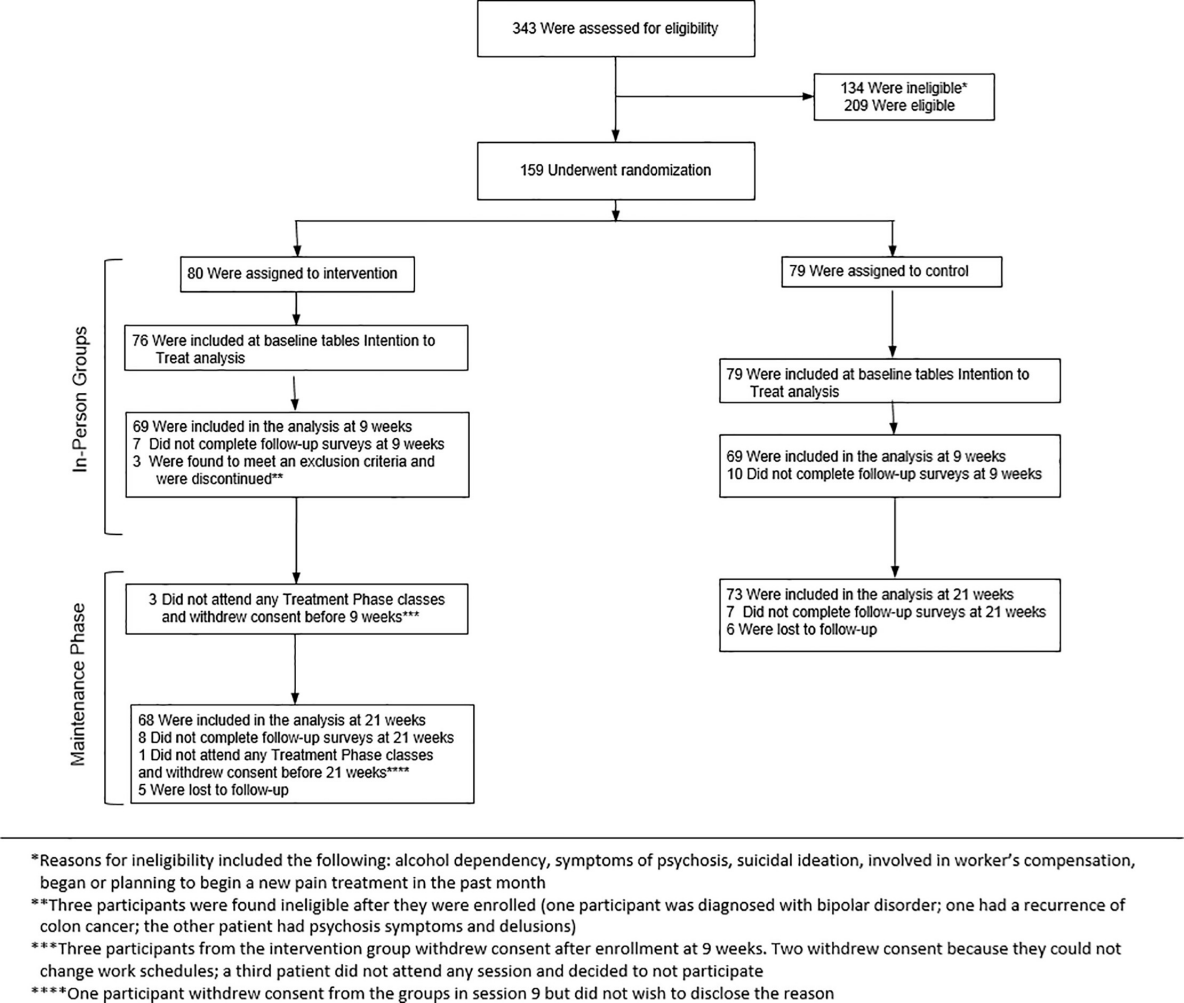
Participant flow in CONSORT diagram.

Screening, eligibility, randomization, and reasons of participants’ ineligibility after enrollment or withdrawal are given in Fig 1.

The recruitment began in November 2014 and finished in October 2016. Three hundred forty-three patients were assessed for eligibility, 209 patients were eligible, and 159 were enrolled and randomized to intervention (80) or control (79).

Four participants in intervention group were excluded from the analysis because they withdrew their consent after being enrolled. A total of 155 participants were included in the ITT analysis and baseline tables. After being enrolled in the study, three participants in the intervention were found to meet an exclusion criterion and were discontinued. These participants were included in the demographic descriptive analysis (Table 1), but not for 9- and 21-week ITT analysis.

**Table 1:** Baseline demographics for participants by group.

Variable	Totals* (N = 155)		Intervention (N = 76)		Control (N = 79)		P-Value
	Mean (SD)		Mean (SD)		Mean (SD)		
Age (22–84)	50.5 (12.3)		50 (12.2)		51 (12.4)		0.62
Gender	N	%	N	%	N	%	
Female	134	86	64	84	70	89	0.42
Male	21	14	12	16	9	11	
Race							
White	29	19	12	16	17	21.5	0.83
Black	87	56	44	58	43	54	
Multiple race	9	6	5	7	4	5	
Unknown or Not Reported	30	30	15	20	16	19	
Ethnicity							
Hispanic	22	14	10	13	12	15	0.71
Non-Hispanic	133	86	66	87	67	85	
Study Sites							
BMC	68	44	33	43	35	44	0.94
DHHC	40	26	19	25	21	27	
CSHC	47	30	24	32	23	29	
Income							
Less than $5K	20	13	9	12	11	14	0.78
$5K-$29.99K	77	50	36	47	41	52	
$30K and over	13	8	6	8	7	9	
Refused/DK/None	45	29	25	33	20	25	
Work Status							
Full/Part time	32	21	15	20	17	22	0.93
Unemployed	22	14	10	13	12	15	
Retired/Home	18	12	9	12	9	11	
Disability	66	42	32	42	34	43	
Other	17	11	10	13	7	9	
Education Level	N	%	N	%	N	%	
< High school/some	27	18	15	20	12	15	0.33
High school degree	53	34	22	29	31	39	
Some college/AA degree	53	34	30	39	23	29	
College degree or >	22	14	9	12	13	17	

*Four withdrew consent to use their data after randomization

### Baseline characteristics

Baseline demographic characteristics are listed in Table 1. Of the 155 participants, the average age was 51 years old, 86% identified as female, 56% identified as black, 36% identified as “other” race, and 19% identified as white. There were no significant differences for baseline characteristics.

Common co-morbidities in the participants were hypertension (41%), obesity (37%), diabetes (23%), insomnia (26%), anxiety (28%), Post-Traumatic Stress Disorder (PTSD) (16%), and any substance use disorder (25%).

The baseline, 9 week, and 21 week measurements of the primary and secondary outcomes are listed in Tables 2–4. At baseline, the average PHQ-9 score for depressive symptoms was 12, which is characterized as moderate depression. Eighty-five percent of participants used pain medication in the last seven days at baseline (opioids: 37%, NSAIDS 48%, other medication: 43%). The average Physical Composite Score (PCS) was 34 and the average Mental Composite Score (MCS) was 36, compared to national average scores of 50. There were significant differences between the intervention and control group for patient activation measure (p = 0.051) and current opioid misuse measures (COMM) (p = 0.0495) at a significant level of 0.1, so they were adjusted for in all subsequent models.

**Table 2:** Baseline outcomes for all participants by intervention and control group.

Variable *(range)* ^	Intervention Baseline (N = 76)		Control Baseline (N = 79)		
	Mean (SD)		Mean (SD)		P-value
Average pain *(0–10)*	7 (1.9)		7 (1.9)		0.97
BPI Interference *(0–10)*	7 (2.2)		6 (2.3)		0.47
BPI Severity *(0–10)*	7 (1.9)		7 (1.8)		0.95
PHQ *(0–27)*	13 (5.6)		11 (5.3)		0.11
Pain Self-Efficacy *(0–60)*	30 (15.4)		32 (13.5)		0.47
Patient Activation Measure *(0–100)*	60 (15.6)		56 (11.8)		0.05*
SF-12 Physical Composite Score *(0–100)*	33 (10.3)		35 (10.6)		0.12
SF-12 Mental Composite Score *(0–100)*	35 (9.5)		36 (10.3)		0.62
Current Opioid Misuse Measure *(0–64)*	11 (5.9)		9 (5.8)		0.05*
	**N**	**%**	**N**	**%**	**P-value**
Pain medication past 7 days	67	88	65	82	0.30
Emergency Department Use	15	21	11	14	0.28

*Significant differences between the intervention and control group for PAM (p = 0.0513), COMM (p = 0.0495) at a significant level of 0.1, so they were adjusted for in all subsequent models.

^Continuous variables are summarized with mean (standard deviation)

**Table 3:** Outcomes for 9 weeks for all participants by intervention and control group.

**Outcomes for Specific Aim 1 (Reduction of Pain)**							
Variable *(range)*	Intervention Baseline (N = 69)		Control Baseline (N = 69)			Total (N = 138)	
	**Mean (SD)**		**Mean (SD)**		**P-value**	**Mean (SD)**	
Average pain *(0–10)*	6 (2.3)		6 (2.0)		0.61	6 (2.1)	
BPI Interference *(0–10)*	6 (2.8)		5 (2.5)		0.64	6 (2.7)	
BPI Severity *(0–10)*	6 (2.2)		6 (2.2)		0.94	6 (2.2)	
**Outcome for Specific Aim 2 (Reduction of Depression)**							
PHQ *(0–27)*	11 (5.5)		10 (5.7)		0.15	10 (5.6)	
**Outcomes for Specific Aim 3 (Increase of Pain Self-Efficacy and Reduced Use of Pain Medication)**							
Pain Self-Efficacy *(0–60)*	36 (15.7)		34.8 (14.1)		0.68	35 (14.8)	
	**n**	**%**	**n**	**%**	**P-value**	**n**	**%**
Pain medication past 7 days	53	77	54	78	0.84	107	78
**Secondary Self Report Outcomes**							
	**Mean (SD)**		**Mean (SD)**		**P-value**	**Mean (SD)**	
Perceived Stress Scale *(0–16)*	7 (3.3)		7 (3.7)		0.85	7 (3.5)	
Patient Activation Measure *(0–100)*	61 (13.6)		62 (16.4)		0.58	62 (15.0)	
SF-12 Physical Composite Score *(0–100)*	36 (10.1)		37 (10.4)		0.68	36 (10.2)	
SF-12 Mental Composite Score *(0–100)*	36 (9.9)		39 (11.4)		0.22	37 (10.7)	
Current Opioid Misuse Measure *(0–64)*	9 (6.4)		8 (6.0)		0.22	9 (6.2)	
	**n**	**%**	**n**	**%**	**P-value**	**n**	**%**
Emergency Department Use	6	8	13	16	0.13	19	13

**Table 4:** Outcomes for 21 weeks for all participants by intervention and control group.

**Outcomes for Specific Aim 1 (Reduction of Pain)**							
Variable *(range)*	Intervention Baseline (N = 68)		Control Baseline (N = 72)			Total (N = 140)	
	**Mean (SD)**		**Mean (SD)**		**P-value**	**Mean (SD)**	
Average pain *(0–10)*	6 (2.0)		6 (2.0)		0.64	6 (2.0)	
BPI Interference *(0–10)*	6 (2.7)		5 (2.7)		0.046*	5 (2.7)	
BPI Severity *(0–10)*	6 (2.3)		6 (2.0)		0.96	6 (2.1)	
**Outcome for Specific Aim 2 (Reduction of Depression)**							
PHQ *(0–27)*	9 (5.4)		10 (5.9)		0.39	10 (5.7)	
**Outcomes for Specific Aim 3 (Increase of Pain Self-Efficacy and Reduced Use of Pain Medication)**							
Pain Self-Efficacy *(0–60)*	34 (14.7)		38 (13.5)		0.10	36 (14.2)	
	**n**	**%**	**n**	**%**	**P-value**	**n**	**%**
Pain medication past 7 days	49	72	60	83	0.11	109	78
**Secondary Self Report Outcomes**							
	**Mean (SD)**		**Mean (SD)**		**P-value**	**Mean (SD)**	
Perceived Stress Scale *(0–16)*	7 (3.3)		7 (3.4)		0.83	7 (3.3)	
Patient Activation Measure *(0–100)*	62 (13.5)		63 (16.2)		0.69	62 (14.9)	
SF-12 Physical Composite Score *(0–100)*	33 (11.0)		38 (9.7)		0.006*	36 (10.6)	
SF-12 Mental Composite Score *(0–100)*	41 (11.6)		38 (11.3)		0.19	40 (11.5)	
Current Opioid Misuse Measure *(0–64)*	9 (6.1)		8 (5.2)		0.28	8 (5.6)	
	**n**	**%**	**n**	**%**	**P-value**	**n**	**%**
Emergency Department Use	9	12	9	11	0.86	18	12

*indicates that the results are statistically significant

In ITT analysis (Table 5), we found no clinically or statistically significant difference between two groups for average pain (RR: 1.03, 95% CI: 0.92, 1.15) or PHQ-9 score (RR: 1.09, 95% CI: 0.92, 1.28) at 9 weeks. For the primary outcomes at 21 weeks, there was no difference of average pain (RR: 0.98, 95% CI: 0.88, 1.08) and PHQ-9 score (RR: 0.89, 95% CI: 0.75, 1.06) for those in the intervention group compared with the control group. Participants who attended the intervention group, there was a 4-point reduction (baseline– 13 points / 21 weeks– 9 points) in PHQ-9 compared with the control group (baseline– 11 points / 21 weeks– 10 points). This translates into a clinically meaningful difference.

**Table 5:** Intention to treat results for outcome data.

	Week 9 Relative Risk (CI)	Week 21 Relative Risk (CI)	RR without time interaction term of 9 week and 21 weeks, if it is not significant
Average painᵖ	1.03 (0.92, 1.15)	0.98 (0.88, 1.08)	1.00 (0.92, 1.08)
BPI Interferenceᵖ	1.00 (0.86, 1.16)	1.17 (0.99,1.37)	1.06 (0.96, 1.18)
BPI Severityᵖ	1.00 (0.89, 1.13)	1.01 (0.90, 1.14)	1.01 (0.92, 1.10)
PHQ-9†	1.09 (0.92, 1.28)	0.89 (0.75, 1.06)	Interaction is significant
Pain Self Efficacy†	1.09 (0.96, 1.25)	0.93 (0.83, 1.05)	Interaction is significant
Patient Activation Measureᵖ	0.99 (0.92, 1.07)	1.00 (0.93, 1.08)	Interaction is significant
SF-12 Physical Composite Scoreᴸ	1.01 (0.92, 1.12)	0.86 (0.77, 0.97)*	Interaction is significant
SF-12 Mental Composite Scoreᴸ	1.01 (0.95, 1.07)	1.07 (1.01, 1.12)*	1.02 (0.96, 1.08)
Current Opioid Misuse Measureᵖ	1.14 (0.92, 1.42)	1.13 (0.91, 1.40)	1.20 (1.10, 1.42)
Pain medication in the last 7 days (Odds Ratio)	0.75 (0.33, 1.68)	0.42 (0.18, 0.98)*	Interaction is significant
ED useᵖ	0.31 (0.10, 0.89)*	0.85 (0.32, 2.22)	0.72 (0.41,1.26)

ᵖ Poisson Model was used for this outcome variable.

† Negative Binomial Model was used for this outcome variable.

ᴸ Log Normal Model was used for this outcome variable.

^OR^ Logistic regression model was use for this outcome variable. The results are OR (95%CI).

* Results are statistically significant and 95% Confidence Intervals (CI) does not include the number 1. All models were adjusted for COMM, PAM and this table shows the “9 week” and “21 week” results. The control group as well as the baseline outcomes were set as reference groups.

For secondary outcomes, at 9 weeks there was no difference in PCS (RR: 1.01, 95% CI: 0.92, 1.12), but was lower at 21 weeks (RR = 0.86 (0.77, 0.97)). At 21 weeks, the intervention group had higher mental quality of life compared with the control group (RR: 1.07, 95% CI: 1.01, 1.12). Although not significant at 9 weeks, at 21 weeks the intervention group had a reduction in any pain medication use compared with the control (OR = 0.42 (0.18, 0.98)). We found that at 9 weeks, the intervention group had fewer ED visits (RR = 0.31 (0.10, 0.89)) (baseline intervention n = 15 reduced to n = 6) (baseline control n = 11 increased to n = 13) compared with control group, but this was not maintained at 21 weeks. There was no meaningful change in the pain severity and pain interreference between the intervention and the control group at 9 and 21 weeks.

### Exploratory attendance to group visits

In terms of attendance, the minimum number of sessions attended was zero and the maximum was ten. The average number of sessions attended was 6.1 (S.D. = 2.9) and the mode is 5. Recorded reasons for no attendance included: lack of transportation, death of family or friends, work conflict, lack of child-care, weather, and doctor’s appointments. The most common reason participants missed a session was that they were “too sick or in “too much pain to come”. Participants in the intervention group who attended few (4 or less) sessions differed from those who had high attendance (Tables A and B in S1 Appendix). They were younger (mean 41 years old), more likely to be female (92%), and reported higher pain, pain severity, and depressive symptoms (PHQ-9 mean = 14.23) than those attending 5 or more groups. Participants in the control group who did not attend a primary care provider appointment during the study (n = 17) were different from the controls who did (n = 62). They were younger (42 years old compared with 54 years old) and used less pain medication than those who did see their PCP.

In the exploratory attendance multivariable regression analysis, we compared intervention participants with different attendance to the control participants who did not visit PCP. Among those who attended 5–6 sessions [RR: 0.80, 95%CI: 0.67, 0.97] or 7–10 sessions [RR: 0.87, 95%CI: 0.76, 1.00, p = 0.05] there was a reduction in average pain. Among participants who attended 5–6 sessions, there was a 28% reduction in PHQ-9 scores at 9 weeks [RR: 0.72, 95% CI: 0.54, 0.97] and a 33% reduction of PHQ-9 scores at 21 weeks [RR: 0.67, 95% CI: 0.47, 0.95] compared to control, which translated into a clinically meaningful result of 4.8 points difference. Participants who attended 7–10 sessions had a 30% reduction in PHQ-9 scores and reduced their opioid use from 42% to 28%.

### Adverse events

Forty-one adverse events occurred in 13 participants in the control group and 19 participants in the intervention group. The most common adverse events were ED visits (11 in the intervention and 17 in the control group). There were two hospital admissions from both the intervention and the control group. Among the 41 adverse events, 40 were determined to be unrelated to the intervention. The one event determined to be due to the intervention was when a participant fell off a swivel chair during a group visit. This participant was not harmed.

### Fidelity and evaluation data

Each group was scored with a monitoring and evaluation checklist by a research assistant for fidelity. The checklist monitored adherence to vitals, centering meditation, delivery of health topic, mind-body activity, and the review of home practice. The maximum possible fidelity score per group is 80. Across the seven cohorts the average fidelity score was 77.3.

## Discussion

This RCT tested the effectiveness of a 21-week mind body self-management medical group visit in a socioeconomic diverse patient population with chronic pain and depressive symptoms and found no different in pain and depressive symptoms compared to primary care visits, with both groups experiencing improvement in symptoms. There was a significant reduction in pain medication use and increase in mental quality of life attributable to the intervention as well as a reduction in total ED visits, reproducing our previous findings of decreased ED utilization [69]. Although the primary outcome of pain and depressive symptoms were not different from a primary care visit, decreased ED use and pain medication use suggest IMGV may be helpful in patients with chronic pain and depression. The study further demonstrates that IMGVs are viable in urban outpatient clinics and CHCs (94% attended at least one IMGV, 72% attended half or more sessions.

Chronic pain places a burden on patients' lives with many patients also suffering from depression [70]. Clinical studies have shown a reduction in depressive symptoms as a secondary outcome [71–74]. In clinical trials on chronic pain and MGVs in low-income patients, our lack of significant reduction in pain is inconsistent with prior studies. For example, Geller et al. conducted a prospective cohort study of MGVs for 42 women in a low-income patient population and showed changes in bodily pain and mental health. [75,76]. Chao et al., conducted a prospective RCT of the effectiveness of a 7-week MGV or an educational booklet control condition in 45 older women with nonspecific chronic pelvic pain [77] and found a reduction in pain intensity immediately following the group sessions [78]. Our study incorporated participants with depressive symptoms and chronic pain, which increases comorbidity; therefore, this was a more difficult population to treat then in previous published studies above.

In the U.S., current treatment options for chronic pain are largely medication-based (opioids) despite mixed evidence of efficacy and increased risk of potentially dangerous side effects, including addiction and death [24,25, 79–83]. In this study, a statistically significant reduction in pain medication use occurred in the intervention compared with the control group. Other MGVS studies have found showed a reduction in pain medication use [71,84].

Chronic pain often leads to poor quality of life and frequent health care utilization [26]. The IMGV showed a significant reduction in ED visits between intervention and control groups at 9 weeks and a non-significant reduction at 21 weeks, suggesting that the active interaction with the clinician at the IMGV may offer opportunities to intervene on subacute issues prior to requiring an ED visit [69]. MGVs have consistently showed a reduction in ED visits, and suggest that the MGVs have the potential to reduce health care costs [85–90]. Literature also supports that pain education, present in this intervention, can contribute to lower healthcare utilization and may provide additional explanation for the reduction in ED visits [91]. The IMGV was helpful to patient’s mental quality of life at 21 weeks, which may be attributable to a reduction in isolation and increase in emotional support [76, 90, 92–93]. The IMGV was protective during the group visit because the participants had access to a clinician and social support.

Based on our previous attendance to clinical group visits, we did not anticipate the variety of different levels of attendance to the IMGV or to the PCP (control). Although asked to visit their PCP clinician, 17 control participants did not attend any PCP visits during the course of the study. Those in the control group who did not visit their PCP during the study were younger and more depressed with higher pain scores, consistent with trends seen elsewhere [94]. Participants who attended few MGVs were clinically different from those who had high attendance. Since low attendance participants had the highest average pain at baseline—this may have been a factor affecting their mobility and ability to attend a minimum number of groups. Not all patients are the right candidates for medical group visits, and it is important to determine who will come to a MGV and who will not [95]. To design the appropriate group intervention, it is important to recognize participants with low attendance and the factors that differentiate them from other participants in the study [96–98].

### Limitations of the study

There are several limitations in this RCT that may have affected the outcomes of the study. For example, it is possible that 9 weeks of active in-person group visits was not long enough to see a significant change when comparing a routine primary care to medical group visit. In addition, at enrollment, some patients may have heightened pain and depressive symptoms, and as time went on, their scores may have regressed to the mean. We also used self-reported measurements for pain and depression, and these can change day-to-day. Another limitation is that we did not have the statistical power to conduct a multi-variable regression for reduction in opioids and NSAIDS because of our small sample size. We performed many analyses and it is possible that the results could appear significant by chance alone. We suggest that these results be taken as suggestive and hypothesis generating for future studies.

In conclusion, MGVs are one way to incorporate patient self-management, non-pharmacological techniques, pain education, and increase social connections into the health care system [99–101]. When comparing groups that attended MGVs or had a PCP visit, both showed a similar reduction in self-reported pain and depressive symptoms. However, our results suggest that IMGV is a feasible adjunct model of care for low-income diverse patients and is more effective than a PCP visit at reducing ED visits and pain medications.

## Supporting information

S1 Appendix Exploratory attendance analysis—Demographic data organized by number of group visit sessions and PCP visits attended. (PDF)

S1 CONSORT Checklist (DOCX)

## Abbreviations

BMC: Boston Medical Center
CHC: Community Health Center
COMM: Current Opioid Misuse Measure
CSHC: Codman Square Health Center
DH: Dothouse Health Center
DSMB: Data Safety Monitoring Board
EBIM: Evidence-Based Integrative Medicine
ECA: Embodied Conversational Agent/ Gabby
ED: Emergency Department
EMR: Electronic Medical Record
MBSR: Mindfulness-Based Stress Reduction
MGV: Medical Group Visits
IMGV: Integrative Medical Group Visits
IRB: Internal Review Board
ITT: Intention to Treat
NSAIDS: Nonsteroidal Anti-Inflammatory Drugs
OWL: Our Whole Lives; an e-health toolkit
PAM: Patient Activation Measure
PCP: Primary Care Provider
PHQ-9: Patient Health Questionnaire– 9 items
PP: Per Protoco
PSEQ: Pain Self-Efficacy Scale
RA: research assistant
RCT: Randomized Controlled Trial
