# Supplementary material for: Effectiveness of integrative medicine group visits in chronic pain and depressive symptoms: A randomized controlled trial
Source: PLoS One. 2019 Dec 18;14(12):e0225540. doi: 10.1371/journal.pone.0225540 (PMC6919581; doi:10.1371/journal.pone.0225540)
Supplement: S1 Appendix — (PDF) [file pone.0225540.s001.pdf]

Table 6: Exploratory attendance analysis - Demographic data organized by number of group visit sessions attended and control group PCP visit at relevant time points (n, %)

Variable	Attended 4 or less sessions (N= 18)	Attended 5-6 sessions (N=22)	Attended 7 or more sessions (N=36)	Attended at least 1 PCP visit (N=62)	Did not attend any PCP visit (N=17)
Age (22-84)*	41 (10.14)	47(10.85)	55 (11.86)	54 (11.66)	42 (10.44)
Gender	N (%)	N (%)	N (%)	N (%)	N (%)
Female	12 (92)	17 (77)	30 (83)	54 (87)	16 (94)
Race					
White	1 (8)	4 (18)	5 (14)	12 (19)	5 (29)
Black	9 (69)	11 (50)	23 (64)	37 (60)	8 (47)
Other	3 (23)	7 (32)	8 (22)	13 (21)	4 (24)
Ethnicity					
Hispanic	3 (23)	1(5)	6 (17)	5 (8)	7 (41)
Non-Hispanic	10 (77)	21(95)	30 (83)	57 (92)	10 (59)
Income					
< than $5K	2 (15)	1 (5)	5 (14)	8 (13)	3 (18)
$5K-$29.99K	7 (54)	11 (50)	16 (45)	34 (55)	7 (41)
$30K and over	1 (8)	2 (9)	3 (8)	6 (10)	1 (6)
Refuse/None	3 (23)	8 (36)	12 (33)	14 (22)	6 (35)
Work Status					
Full/Part time	3 (23)	3 (14)	8 (22)	11 (18)	6 (35)
Unemployed	2 (15)	2 (9)	5 (14)	9 (14)	3 (18)
Retired	1 (8)	0 (0)	8 (22)	7 (11)	2 (12)
Leave/Disability	5 (39)	13 (59)	11 (31)	29 (47)	5 (29)
Other	2 (15)	4 (18)	4 (11)	6 (10)	1 (6)
Education Level					
< high school	2 (15)	5 (23)	5 (14)	6 (10)	6 (35)
HS degree	5 (38)	6 (27)	11 (30)	26 (42)	5 (29)
Some college	5 (38)	8 (36)	15 (42)	20 (32)	3 (18)
College	1 (8)	3 (14)	5 (14)	10 (16)	3 (18)

*Age is summarized with mean (standard deviation)

Table 7: Descriptive Data for Exploratory Dose Analysis Outcomes for Specific Aims Outcomes

Variable	Total N=155	Intervention			Control	
		Attended 4 or less sessions (N= 18)	Attended 5-6 sessions (N=22)	Attended 7 or more sessions (N=36))	Attended at least 1 PCP Appt. (N=62)	Not attend any PCP Appt. (N=17)
Baseline Average pain*	6.99 (1.90)	7.38 (2.10)	6.64 (1.99)	6.89 (1.83)	6.98 (1.89)	7.00 (1.97)
9 weeks (Wk) Average pain*	6.21 (2.15)	7.00 (2.45)	6.00 (2.31)	6.28 (2.20)	6.27 (2.07)	5.46 (1.85)
21 Wk Average pain*	6.26 (1.97)	6.45 (2.16)	5.76 (2.02)	6.34 (1.89)	6.15 (2.05)	7.15 (1.46)
Baseline PHQ-9*	12.12 (5.47)	14.23 (4.40)	12.82 (4.85)	11.83 (6.27)	11.18 (5.44)	12.29 (4.78)
9 Wk PHQ-9*	10.32 (5.63)	14.55 (3.33)	9.77 (4.98)	10.69 (5.98)	8.95 (5.68)	12.54 (5.08)
21 Wk PHQ-9*	9.71 (5.66)	11.82 (5.34)	8.05 (5.57)	9.14 (5.23)	9.73 (5.91)	12.08 (5.51)
Baseline Pain Self Efficacy*	30.94 (14.41)	32.77 (15.18)	28.27 (16.43)	30.08 (15.48)	32.31 (13.93)	29.76 (11.87)
9 Wk Pain Self Efficacy*	35.30 (14.85)	30.91 (15.35)	38.86 (14.27)	35.47 (16.58)	35.16 (14.68)	33.08 (11.37)
21 Wk Pain Self Efficacy*	36.00 (14.21)	31.00 (13.34)	35.05 (13.31)	34.03 (16.24)	37.95 (13.45)	38.23 (14.12)
	N (%)	N (%)	N (%)	N (%)	N (%)	N (%)
Baseline Pain Medication use	132 (85)	13 (100)	19 (86)	31 (86)	52 (84)	13 (76)
9 Wk Pain Medication use	107 (78)	8 (73)	19 (86)	26 (72)	43 (77)	11 (85)
	N (%)	N (%)	N (%)	N (%)	N (%)	N (%)
21 Wk Pain Medication use	108 (78)	9 (82)	14 (67)	25 (71)	50 (85)	10 (77)
Baseline NSAID use	74 (48)	7 (54)	11 (50)	19 (53)	33(49)	4 (24)
9 Wk NSAID use	66 (43)	5 (38)	13 (59)	18 (50)	23 (34)	7 (41)
21 Wk NSAID use	65 (42)	7 (54)	9 (41)	15 (42)	26 (39)	8 (47)
Baseline opioid use	57 (37)	6 (46)	10 (45)	15 (42)	22 (35)	4 (24)
9 Wk opioid use	40 (26)	3 (23)	9 (41)	10 (28)	18 (29)	4 (24)
21 Wk opioid use	52 (34)	7 (54)	10 (45)	10 (28)	21 (34)	4 (24)
Baseline Other Pain Medication	67 (43)	8 (62)	7 (32)	10 (28)	34 (51)	8 (47)
9 Wk Other Pain Medication	59 (38)	7 (54)	5 (23)	14 (39)	28 (42)	5 (29)
21 Wk Other Pain Medication	58 (37)	8 (62)	4 (62)	10 (28)	30 (45)	6 (35)

*Continuous variables are summarized with mean (standard deviation)
